# Quantification of the Relationship of Pyridoxine and Spirometry Measurements in the United States Population

**DOI:** 10.1016/j.cdnut.2023.100078

**Published:** 2023-07-13

**Authors:** Alexander A. Huang, Samuel Y. Huang

**Affiliations:** 1 Cornell University, Ithaca, New York; 2 Northwestern University Feinberg School of Medicine, Chicago, Illinois; 3 Virginia Commonwealth University School of Medicine, Richmond, Virginia

**Keywords:** vitamin B6, spirometry, regression, spline, NHANES

## Abstract

**Background:**

There has been evidence to suggest associations between vitamins and lung function.

**Objective:**

This study aimed to examine the association between vitamin B6 and spirometry values.

**Methods:**

A cross-sectional study was done using National Health and Nutritional Examination Surveys (NHANES) 2007–2012, which is a nationally representative, modern cohort. Spirometry, a clinical pulmonary function test, measured the amount and speed of air a person could exhale after taking the deepest possible breath after forceful expiratory volume at 1 s (FEV1) and forced vital capacity (FVC). After determination of the relationship of the linearity of variables, univariable and multivariable models were fitted to investigate the effect of vitamin B6 on FEV1 and FVC. The National Center for Health Statistics Ethics Review Board granted permission for the study’s data collection and analysis.

**Results:**

Of 19,160 individuals who had complete information on vitamin B6 intake, FEV1, and FVC, it was found each mg of vitamin B6 intake was associated with increase in 166.41 mL of FEV1 (95% CI: 156.71, 176.12; *P <* 0.01) and 221.6 mL of FVC (95% CI: 209.62, 233.57; *P <* 0.01). After controlling for potential confounders (age, race, sex, body mass index, education, and income), multiple linear regression found that each mg of vitamin B6 was associated with increase in 25.98 mL of FEV1 (95% CI: 19.15, 32.80, *P <* 0.01) and 38.97 mL of FVC (95% CI: 30.65, 47.30, *P <* 0.01).

**Conclusion:**

Increased vitamin B6 intake is associated with improvement in lung function. Further prospective studies are required to ascertain whether increased vitamin B6 can lead to increased long-term spirometry measurements and the specific therapeutic dose–response relationship.

## Introduction

Lung function is crucial for overall health, as it affects daily activities and physical and mental well-being [[1], [2], [3]]. There is a push toward research that focuses on understanding factors that impact respiratory health and how they lead to changes in lung function [4,5]. Improving lung function leads to better daily activity, increased energy, and mental well-being and reduces risk of chronic respiratory conditions like chronic obstructive pulmonary disease (COPD) [[6], [7], [8]]. Finding therapeutics and interventions that improve lung function is an area of active research [9].

Vitamins’ anti-inflammatory and antioxidative properties have been proposed to beneficially influence the oxidative and inflammatory status of individuals [10]. Specifically, vitamin B6 is a water-soluble nutrient that can be found in both animal and plant-based foods in the forms of pyridoxal, pyridoxine, and pyridoxamine [11]. After absorption, the liver converts vitamin B6 into its biologically active form, PLP, which accounts for approximately 60% of the circulating vitamin B6 in the body [12]. PLP acts as a cofactor in various metabolic and immune system processes [13].

There is current research that shows an inverse relationship between levels of vitamin B6 intake and inflammation [14]. Individuals with low vitamin B6 exhibit higher levels of inflammatory markers, which are thought to be from the fact that vitamin B6’s active form, PLP, is drawn to sites of active inflammation [15]. Vitamin B6 is a key player in the antioxidant system and stops lipid peroxidation [16]. There are ongoing studies showing vitamin B6’s roles in signaling pathways that involve NF-кB, NLRP3-mediated caspase-1 activation, and AMPK phosphorylation, which have implications on lung function [11]. There have been few studies on vitamin B6 and lung function. The goal of this study is to examine the potential antioxidant and anti-inflammatory effects of vitamin B6 on lung function through spirometry measurements from a large, nationally representative sample of US adults with data from the NHANES 2007–2012 cohort with spirometry data.

## Methods

We performed a cross-sectional cohort study from publicly available data from the 2007–2012 NHANES. The methods behind acquisition and analysis of the data are described by the National Center for Health Statistics, approved by the National Center for Health Statistics Ethics Review Board [17].

### Dataset and cohort selection

For the study, we utilized the National Health and Nutritional Examination Surveys 2007-2012 (NHANES) data and included individuals (*n* = 19,160) that had complete data on daily nutritional questionnaires, forced expiratory volume at 1 second (FEV1), and forced vital capacity (FVC).

### Assessment of the dependent variables FEV1 and FVC

Spirometry is a standard test for measuring lung function by evaluating the amount and speed of air exhaled after taking a deep breath. The test provides information on lung volume and air flow rates, helping diagnose respiratory disorders like asthma and COPD. The Ohio 822/827 dry-rolling seal volume spirometer was used to measure the FEV1 and the FVC as dependent variables. Participants were instructed to perform maximal forceful exhalations in a standing position, unless physically restrained. A nose clip was placed on their nose to prevent air leakage and they were asked to raise their chin and slightly extend their neck. They were instructed to take a deep breath, place the mouthpiece in their mouth to form a tight seal, and exhale as hard and fast as possible for at least 6 seconds [18]. Participants continued the spirometry until repeatable and acceptable results were obtained according to the criteria set by the American Thoracic Society [19].

### Independent variable

During the study, participants were questioned about their dietary habits, with a focus on their food, beverage, and supplement consumption [20]. This information was transformed into daily nutrient intake figures, and vitamin B6 intake was extracted. The total vitamin B6 intake was calculated by adding up all sources of the nutrient, including food, beverages, and supplements.

### Model construction and statistical analysis

Descriptive statistics for all patients were computed for relevant demographic covariates and vitamin B6 intake (mg/d). Comparison between the variables was done using a chi-square test for categorical variables and *t* tests for continuous variables. The effect of vitamin B6 intake on symptoms of depression was initially assessed in univariable models. Multivariable models were then constructed to evaluate the impact of vitamin B6 intake after adjusting for age, race, sex, income to poverty ratio, BMI, and degree of schooling.

### Choosing how to model vitamin B6

Using an ANOVA, the relationship between the restricted cubic spline and linear logistic model was compared to see if there was a curvilinear relationship between vitamin B6 intake and spirometry measurements. The model with the best fit was utilized. Based on the graphical representation and the Food and Nutrition Board’s (FNB) evidence, a simplified piecewise function with a cut-point would be established if the univariable restricted cubic spline displayed a curvilinear relationship. The original restricted cubic spline and the piecewise function were compared using ANOVA to see if there was any information lost. If there was no significant difference in the model fit, the piecewise function was used, while the restricted cubic spline was used if it was the better model. If the *P* value was less than 0.05, the results were deemed statistically significant [21].

## Results

Table 1 describes demographic variables including important covariates in the NHANES 2007–2012 cohort with complete FEV1, FVC, and daily nutritional values questionnaire (*n* = 19,160). This cohort contained 9521 females (50%) and 9639 Males (50%). The mean age was 35.22 (SD=21.12), with 7415 (39%) non-Hispanic White participants, 4412 (23%) non-Hispanic Black participants, 3563 (19%) Hispanic participants, and 3770 (20%) participants of another race. The mean vitamin B6 intake was 1.96 (SD=1.38) mg/d.

**TABLE 1:** Patient demographics and nutrition covariates

Total no. of patients	19,160
FEV1 (mL)	2902.76 (970.26)
FVC (mL)	3609 (1202.42)
Age (y)	35.22 (21.12)
Sex	
Female	9521 (0.5)
Male	9639 (0.5)
Race/ethnicity	
Non-Hispanic White	7415 (0.39)
Non-Hispanic Black	4412 (0.23)
Hispanic	3563 (0.19)
Other	3770 (0.2)
BMI (kg/m^2^)	26.62 (7.42)
Education	
Less than high school	1253 (0.1)
High school	2922 (0.23)
Some college	3741 (0.29)
College graduate	2903 (0.23)
Other	1991 (0.16)
Ratio of income to poverty	
<1	4468 (0.25)
≥1	13,175 (0.75)
Vitamin B (mg)	1.96 (1.38)

Descriptive statistics for current cohort. Values in brackets are standard deviations for continuous variables and proportions for categorical variables.

FEV1, forced expiratory volume at 1 second; FVC, forced vital capacity.

**FIGURE 1:**
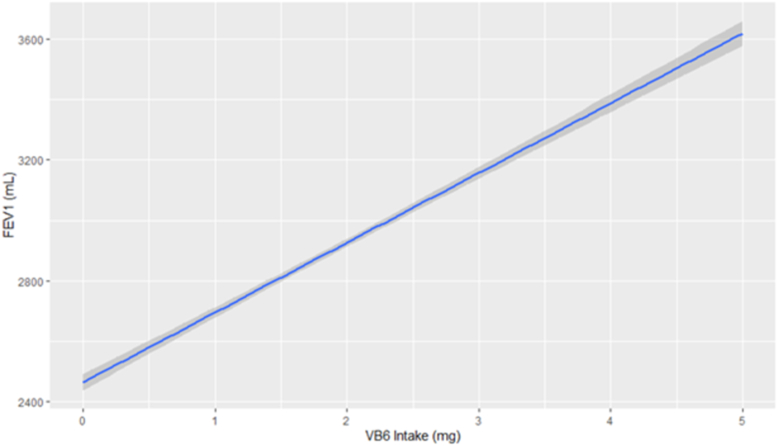
Relationship between vitamin B6 and FEV1. Relationship between vitamin B6 (x-axis, ascertained through dietary journals) and FEV1 (y-axis, ascertained through spirometry measurements). FEV1, forced expiratory volume at 1 sec.

A univariable restricted cubic spline model was fitted for FEV1 on vitamin B6 as a univariable model to test for linearity. The restricted cubic spline was compared to the linear regression model via an ANOVA and showed the restricted cubic spline to be no different than the linear model. Figure 1, which is the linear regression model, shows an increasing effect of FEV1 for each mg of vitamin B6 intake. In the univariable model of vitamin B6 on FEV1, for each 0.1 mg of vitamin B6, there was an increase of 166.41 mL on FEV1 (95% CI: 156.71, 176.12; *P <* 0.01).

**FIGURE 2:**
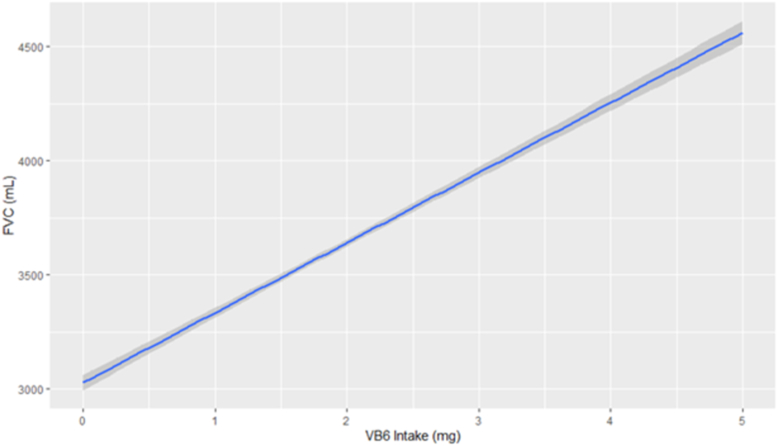
Relationship between pyridoxine and FVC. Relationship between pyridoxine (x-axis, ascertained through dietary journals) and FVC (y-axis, ascertained through Spirometry measurements). FVC, forced vital capacity.

A univariable restricted cubic spline model was then fitted for FVC on vitamin B6 as a univariable model to test for linearity. The restricted cubic spline was compared to the linear regression model via an ANOVA and showed the restricted cubic spline to be no different than the linear model. Figure 2, the linear regression model, shows an increasing effect of FVC for each mg of vitamin B6 intake. The univariable model of vitamin B6 on FVC shows for each mg of vitamin B6 there was an increase of 221.6 mL on FEV1 (95% CI: 209.62, 233.57; *P <* 0.01).

Table 2 reports the results for the multivariable model for vitamin B6 on FEV1 after controlling for age, race, sex, BMI, degree of schooling, and income to poverty ratio. Even after controlling for confounders, each mg of increased Vitamin B6 is associated an increase of 25.98 mL on FEV1 (95% CI: 19.15, 32.18; *P <* 0.01). Table 3 reports the results for the multivariable model for vitamin B6 on FVC after controlling for age, race, sex, BMI, degree of schooling, and income to poverty ratio. Even after controlling for confounders, each mg of increased vitamin B6 is associated an increase of 38.97 mL on FVC (95% CI: 30.65, 47.3; *P <* 0.01).

**TABLE 2:** Multivariable model for vitamin B6 on FEV1

	Estimate	95% CI (2.5%, 97.5%)	*P*
Vitamin B6	25.98	(19.15, 32.8)	<0.01
Male	925.29	(905.32, 945.27)	<0.01
Age	-31.26	(-31.88, -30.64)	<0.01
Non-Hispanic Black	-320.85	(-354.89, -286.81)	<0.01
Non-Hispanic White	90.92	(60.16, 121.68)	<0.01
Other Hispanic	-59.30	(-98.74, -19.86)	<0.01
Other race–including multiracial	-333.82	(-383.64, -293.99)	<0.01
BMI	-4.67	(-6.12, -3.23)	<0.01
9-11th grade (includes 12th grade)	26.00	(-29.56, 81.55)	0.36
College graduate or above	219.18	(163.1, 275.25)	<0.01
High school graduate/GED or equivalent	65.97	(5.16, 126.77)	0.03
Less than 9th grade	28.26	(-52.97, 109.48)	<0.01
Some college or AA degree	126.31	(73.18, 179.44)	<0.01
Income poverty ratio	40.96	(34.21, 47.71)	<0.01

AA, Associate in Arts; FEV1, forceful exhalation at 1 second; GED, General Educational Development test.

**TABLE 3:** Multivariable model for vitamin B6 on FVC

	Estimate	95% CI (2.5%*,* 97.5%)	*P*
Vitamin B6	38.97	(30.65, 47.3)	<0.01
Male	1289.69	(1265.34, 1314.05)	<0.01
Age	-29.18	(-29.94, -28.42)	<0.01
Non-Hispanic Black	-343.67	(-385.17, -302.16)	<0.01
Non-Hispanic White	279.99	(242.48, 317.49)	<0.01
Other Hispanic	-64.30	(-112.4, -16.21)	0.01
Other race–including multiracial	-415.06	(-469.72, -360.4)	<0.01
BMI	-9.99	(-11.75, -8.24)	<0.01
9-11th grade (includes 12th grade)	68.16	(0.42, 135.91)	0.05
College graduate or above	254.01	(185.63, 322.38)	<0.01
High school graduate/GED or equivalent	91.54	(26.17, 156.91)	0.01
Less than 9th grade	7.40	(-91.64, 106.45)	0.88
Some college of AA degree	149.94	(85.15, 214.72)	<0.01
Income poverty ratio	44.64	(36.41, 52.87)	<0.01

AA, Associate in Arts; FVC, forced vital capacity; GED, General Educational Development test.

## Discussion

In this large cross-sectional, cohort study of adults in the United States, we found that participants with higher vitamin B6 intake had higher spirometry values for FEV1 and FVC. Even after controlling for potential confounding variables (age, sex, race, BMI, education, and income to poverty ratio), these associations remained significant.

Many studies have sought to quantify the association between respiratory diseases that impact FEV1 and FVC and vitamins. Some of these diseases are asthma, COPD, and interstitial lung disease (ILD) [[22], [23], [24], [25], [26]]. A meta-analysis performed by Tsiligianni and van der Molen [27] showed various vitamins (including C, D, E, and A) that have been shown to reduce COPD exacerbations and suggested high vitamin intake can reduce declines in FEV1.

The findings of our study are novel given the fact that there is a complete paucity of studies linking the association between vitamin B6 and decreased lung function. Most studies are old or severely underpowered. In 1993, Sur et al. [28] performed a double-blind, placebo-controlled trial in which 300 mg of pyridoxine were given to steroid-dependent asthmatics (*n* = 31) and found no difference in various metrics including FEV1 between the 2 groups. Cheng et al. [29] did a nationally representative cross-sectional study with patients with COPD and found higher vitamin B6 intake was associated with a 20% lower frailty risk. Hodges et al. [30] found that in females with cystic fibrosis, there was decreased vitamin B6 intake, and in that subset, they had lower gastrointestinal complaints compared to females without cystic fibrosis.

There have been biological models suggesting that vitamin B6 has an anti-inflammatory effect on the lungs. Bai et al. [31] performed a study with different concentrations of B vitamins including vitamin B6 and found that the B vitamins could block lung injury from exposure to toxins and even regulate expression on the level of transcription to protect against insults to the lung [31,32]. In 1984, Moriguchi et al. [33] found that rats that were pyridoxine deficient had decreased phagocytic function of alveolar macrophages and macrophage activating factors, which are responsible for protecting lung function. More recently, in 2020 Du et al. [11] found that vitamin B6 can act on the sphingosine-1-phosphate pathway and reduce inflammation by acting on 2 major pathways of inflammation, the NFκB and mitogen-activated protein kinase signaling pathways.

### Strengths

The sample, the method of analysis, the control over confounders, and the dependent variable are among our study’s primary strengths. We used a large, powerful sample that was representative of the adult population in the United States. Using recommendations from the FNB, our method of analysis outlined flexible model selection to adjust for the connection between FEV1 and FVC and vitamin B6. To confirm the independence of the relationship between depression symptoms and vitamin B6, significant confounders were appropriately added. Spirometry, a clinically utilized tool for assessing lung function was done carefully in accordance with standards and guidelines set forth by the American Thoracic Society.

### Limitations

Due to their inability to determine the causality or directionality of the variables, as well as their inability to determine temporality, cross-sectional studies are subject to biases caused by recall and misclassification. The study also provides little insight into whether having low levels of vitamin B6 is associated with decreased lung function on an acute or chronic basis. The amounts of vitamin B6 consumed are estimated based on nutrient files recalled by the individual and classified by surveyors. As a result, it is possible that these estimates do not reflect the true intake of vitamin B6.

In conclusion, increased vitamin B6 intake is associated with increased lung function as measured by FEV1 and FVC.

## Author contributions

The authors’ responsibilities were as follows – AH, SH: design of the study, writing, data analysis, and final content; and both authors: read and approved the final manuscript.

## Conflict of interest

The authors reported no conflicts of interest.

## Funding

The authors reported no funding received for this study.

## Data availability

Data described in the manuscript is publicly and freely available without restriction at the NHANES section of the CDC website: https://wwwn.cdc.gov/nchs/nhanes/Default.aspx. alexander.huang@northwestern.edu.

## Declaration of interests

The authors declare that they have no known competing financial interests or personal relationships that could have appeared to influence the work reported in this paper.
